# Gastrointestinal peptides in children before and after hematopoietic stem cell transplantation

**DOI:** 10.1186/s12885-020-06790-9

**Published:** 2020-04-15

**Authors:** Szymon Skoczeń, Magdalena Rej, Kinga Kwiecińska, Danuta Pietrys, Przemysław J. Tomasik, Małgorzata Wójcik, Wojciech Strojny, Agnieszka Dłużniewska, Katarzyna Klimasz, Kamil Fijorek, Michał Korostyński, Marcin Piechota, Walentyna Balwierz

**Affiliations:** 1 Department of Oncology and Hematology, University Children’s Hospital in Krakow, Jagiellonian University Medical College, Wielicka St. 265, 30-663 Krakow, Poland; 2 Department of Oncology and Hematology, University Children’s Hospital in Krakow, Wielicka St. 265, 30-663 Krakow, Poland; 3 Department of Clinical Biochemistry, University Children’s Hospital in Krakow, Jagiellonian University Medical College, Wielicka St. 265, 30-663 Krakow, Poland; 4 Department of Pediatric and Adolescent Endocrinology, University Children’s Hospital in Krakow, Jagiellonian University Medical College, Wielicka St. 265, 30-663 Krakow, Poland; 5 Stem Cell Transplantation Center, University Children’s Hospital in Krakow, Wielicka St. 265, 30-663 Krakow, Poland; 6 Department of Biochemistry, University Children’s Hospital in Krakow, Wielicka St. 265, 30-663 Krakow, Poland; 7 Department of Statistics, Cracow University of Economics, 27 Rakowicka Str., 31-510 Krakow, Poland; 8 Department of Molecular Neuropharmacology, Institute of Pharmacology of Polish Academy of Sciences, 12 Smętna St., 31-343 Krakow, Poland

**Keywords:** Hematopoietic stem cell transplantation, Peptides regulating gastrointestinal tract functions, Children

## Abstract

**Background:**

Gastrointestinal tract function and it’s integrity are controlled by a number of peptides whose secretion is influenced by severe inflammation. In stomach the main regulatory peptide is ghrelin. For upper small intestine cholecystokinin and lower small intestine glucagon-like peptide- 1 are secreted, while fibroblast growth factor-21 is secreted by several organs, including the liver, pancreas, and adipose tissue [12]. Hematopoietic stem cell transplantation causes serious mucosal damage, which can reflect on this peptides.

**Methods:**

The aim of the study was to determine fasting plasma concentrations of ghrelin, cholecystokinin, glucagon- like peptide-1, and fibroblast growth factor-21, and their gene expressions, before and 6 months after hematopoietic stem cell transplantation.27 children were studied, control group included 26 healthy children.

**Results:**

Acute graft versus host disease was diagnosed in 11 patients (41%, *n* = 27). Median pre-transplantation concentrations of gastrointestinal peptides, as well as their gene expressions, were significantly lower in studied group compared with the control group. Only median of fibroblast growth factor-21 concentration was near-significantly higher before stem cell transplantation than in the control group. The post–hematopoietic transplant results revealed significantly higher concentrations of the studied peptides (except fibroblast growth factor-21) and respective gene expressions as compare to pre transplant results. Median glucagone like peptide-1 concentrations were significantly decreased in patients with features of acute graft versus host disease. Moreover, negative correlation between glucagone like peptide-1 concentrations and acute graft versus host disease severity was found.

**Conclusions:**

Increased concentrations and gene expressions of gastrointestinal tract regulation peptides can be caused by stimulation of regeneration in the severe injured organ. Measurement of these parameters may be a useful method of assessment of severity of gastrointestinal tract complications of hematopoietic stem cell transplantation.

## Background

Impaired intestinal function is a common complication of hematopoietic stem cell transplantation (HSCT). Damage to the gastrointestinal (GI) mucosa in patients undergoing HSCT is a serious but still poorly understood complication. Toxicity of HSCT conditioning regimens and graft-versus-host disease (GvHD) result in a 5-fold increase of the risk of significant GI complications compared with other cancer survivors [1, 2]. Chemotherapy and total body irradiation (TBI) can damage GI mucosa and cause diffuse inflammation of GI tract. This leads to disruption of integrity of GI mucosa with subsequent transfer of bacterial lipopolysaccharides and other danger/pathogen-associated molecular patterns (DAMPs/PAMPs) into the circulation [3]. The intestine is also known as the largest endocrine organ in the body. It strongly influences other organs, including the brain via the gut-brain axis [4]. The majority of GI regulatory peptides are secreted by strictly defined sections of the intestine [5]. Ghrelin is produced mainly in the stomach by P/D1 cells, cholecystokinin (CCK) is secreted mainly by the I cells of the upper small intestine, while glucagone like peptide-1 (GLP-1) is produced by the endocrine L cells in the lower intestine [6–11]. Fibroblast growth factor-21 (FGF21) is secreted by several organs, including the liver, pancreas, and adipose tissue [12]. The intensity of GI dysfunction can be assessed using mucositis grading and parenteral nutrition requirements, but these tools cannot identify the most severely affected parts of the GI tract [13]. Endoscopy is rarely performed in the early post-HSCT phase due to the high risk of severe complications. In addition, the test load with nutrients is unreliable in this phase. Due to the differences in the anatomic distribution of intestinal endocrine cells, studies of alterations in GI peptide concentrations might help to localize the affected sections of the gut and assess the severity of inflammation. Thus, there is a need to identify simple and noninvasive tests that can assess the location and severity of gut damage. Additional comparison of marker concentrations before and several months after HSCT can explain the mechanisms of destruction and restoration of the GI tract [14–16]. The aim of this study was to determine and analyze the selected GI peptides secreted on different levels of the gut in patients before and after HSCT.

## Methods

### Study groups

A group of 27 children aged 1.5–19 years (median 9.6 years) was referred to the Stem Cell Transplantation Centre of the University Children’s Hospital in Krakow and was included in this study. One patient of 19 years old started the treatment being underage and remain for the treatment and the observation in Children Hospital being over 18, therefore fulfilled inclusion criteria of the study. The patients were assessed twice—before HSCT (pre-HSCT group) and approximately 6 months after HSCT (post-HSCT group). Diseases that were the indication for HSCT are listed in Table 1. Patients with malignancies, except for juvenile myelomonocytic leukemia (JMML), were referred for HSCT in complete remission. Characteristics of the transplantation procedures are detailed in Table 2.

**Table 1:** Indications for HSCT (pre-HSCT group)

Diagnosis	Number (%)
Acute lymphoblastic leukemia (ALL)	11 (40.7)
Acute myeloblastic leukemia (AML)	4 (14.8)
Chronic myelocytic leukemia (CML)	1 (3.6)
Myelodysplastic syndrome (MDS)	1 (3.6)
Juvenile myelomonocytic leukemia (JMML) and AML	1 (3.6)
**Neoplastic diseases – total**	**18 (70)**
Severe aplastic anemia (SAA)	4 (14.8)
Chronic granulomatous disease (CGD)	3 (8)
Autoimmune lymphoproliferative syndrome (ALPS)	1 (3.6)
Hyper IgM syndrome (HIgM)	1 (3.6)
**Non-neoplastic diseases – total**	**9 (30)**

**Table 2:** Types of HSCT procedures

Type of HSCT	n (%)	Disease (n)
Allogeneic n = 27 (100%)	MUD - 16 (59)	ALL – 8
		AML - 4
		CML - 1
		SAA - 1
		CGD – 2
	MSD - 9 (33)	ALL – 3
		SAA - 2
		JMML and AML - 1
		CGD - 1
		HIgM - 1
		MDS - 1
	MFD - 2 (8)	SAA – 1
		ALPS - 1

*ALL* acute lymphoblastic leukemia

*ALPS* autoimmune lymphoproliferative syndrome

*AML* acute myeloblastic leukemia

*CGD* chronic granulomatous disease

*CML* chronic myelocytic leukemia

*HIgM* hyper IgM syndrome

*JMML* juvenile myelomonocytic leukemia

*MDS* myelodysplastic syndrome

*SAA* severe aplastic anemia

In more than half of the patients (16 patients, *n* = 27) a conditioning regimen was based on Busulfan/Treosulfan. Total body irradiation (TBI) was used in 7 of patients, 4 patients received regimen based on Cyclofsphamide. Most patients (85%) in whom graft-versus-host disease (GvHD) prophylaxis was used received methotrexate combined with cyclosporine. Mucositis was diagnosed in 82% cases (22 patients), grade III and IV mucositis in 26% (7 patients). The key clinical data of the HSCT recipients are presented in Table 3. Mucositis requiring parenteral nutrition was found in almost half (48%) of the patients. Systemic glucocorticoids were used in 19 children in the post-HSCT group to treat complications of HSCT. In 11 of patients aGvHD was seen, including intestinal involvement in one. According to the aGvHD grader (agvhd.com), grade II and III aGvHD was found in 22% cases (6 patients). In two cases multiple locations of aGvHD occurred (II/C - skin+liver, III/C - skin+GI + liver). The patients with aGvHD were treated with additional immunosuppressive agents, including tacrolimus, mycophenolate mofetil, and etanercept. Six months after HSCT, four children still received tapered doses of immunosuppressive agents other than glucocorticoids. The control group consisted of 11 boys and 15 girls aged 4.3 to 16.0 years **(**median 12.2 years). The control children were recruited among family donors, siblings of patients treated with HSCT, and unrelated healthy children. They all had negative medical history, no signs or symptoms of acute or chronic diseases, and no abnormalities in laboratory tests (CBC, serum ALT, and creatinine levels).

**Table 3:** Characteristics of HSCT recipients

Number of patients	27
Sex	boys-20, girls-7
Age (years)	1.5–19 (mean 9, median 9.6)
Neoplastic diseases, n (n %)	18 (67%)
Chemotherapy before HSCT, n (n %)	17 (63%)
Local radiotherapy	5 (CNS-4,Testes-1)
Time since diagnosis (years)	
Neoplastic diseases	Median-1, mean-2; range 0.1–7
Non-neoplastic diseases	Median-1.5, mean-3.8, range 0.1–13
Conditioning regimen based on busulfan or treosulfan, n (n %)	16(60%)
Total body irradiation – 12Gy/6fractions, n (n %)	7 (26%)
GvHD prophylaxis, n (n %)	
CSA	4 (15%)
MTX + CSA	23 (85%)
Mucositis, n (%)	22 (82%)
Grade, n	I-7, II-8, III-6, IV-1
Intravenous alimentation due to mucositis (%)	48
aGvHD, n (n %)	11 (41)
Localisation, %	Gut-9, Skin-91, Liver-27
Grade, n	IA-1, IB-4, IIB-1, IIC-3, IIIC-2
Systemic glucocorticoid treatment, n (%)	19 (70)
Systemic glucocorticoid treatment (days)	Median-3.5, mean-3.6; range 0.1–11
Time from discontinuation of systemic glucocorticoids to the second assessment (months)	Median-3.6, mean-4.5; range 0.5–14
Time from discontinuation of immunosuppressive treatment to the second assessment (months)	Median 1.6; range 0–9
Time from HSCT to the second assessment (months)	Median 6.3 (5.9–19.1)

*aGvHD* acute graft-versus-host disease, *CSA* cyclosporin, *MTX* methotrexate

### Anthropometric measurements

Height and body weight measurements were performed by an anthropometrist. Body mass index (BMI), BMI percentile (BMIPerc) and BMI SDS (BMISDS) were calculated using online WHO BMI calculators [17]. The results were compared to regional reference values, and the reference values were published by the WHO [17–19]. The BF mass and BF% were measured using bioimpedance and calculated according to the method described by Kushner RF and Schoeller DA [20].

### Study protocol

Fasting blood samples were collected in the morning. Patients treated with HSCT were assessed immediately before conditioning and after a median of 6.3 months after HSCT. In the control group samples were obtained once, after enrollment to the study. Blood samples were collected in EDTA and aprotinin tubes, (Becton-Dickinson; UK), and tubes with no anticoagulants. The tubes were delivered to the laboratory immediately and centrifuged for 15 min at 3000 rpm using a horizontal rotor. Serum and plasma samples were stored at -80 °C until the time of measurement. Subsequently, mononuclear cells were separated for microarray followed by total RNA extraction.

### Laboratory measurements

Plasma concentrations of the peptides were measured using EIA kits: ghrelin, CCK, GLP-1 (Phoenix Pharmaceuticals, Inc., USA), and FGF-21 (Millipore Corporation, USA). The sensitivity of the methods are provided by kit suppliers and are as follows: ghrelin – 0.08 ng/ml (14% intra- assay and 5% interassay variability),CCK – 0.06 ng/ml (5% intra- assay and 9% interassay variability), GLP-1 – 0.18 ng/ml (14% intra- assay and 5% interassay variability), FGF-21 – 0.016 ng/ml (5.8% intra- assay and 9% interassay variability).

### Microarray analysis

Microarray analysis used a GeneChip Human Gene 1.0 ST Arrays (Affimetrix, Santa Clara, USA) according to the manufacturer’s protocol. GLP-1 expression data were not available in the Affimetix database, and thus we checked the results of GLP-1 receptor gene expression. Gene loci and Affimetrix codes of the tested peptides are presented in Table 4.

**Table 4:** Comparison of mean parameters and standard deviation of genes expression of peptides regulating gastrointestinal tract. *P*-values after ANOVA and Benjamini-Hochberg correction (B-H) are provided

Gene	Gene locus	Affimetrix code	Expression		p/ B-H
Ghrelin	3p26-p25	8,085,293	post-HSCT 6.84 ± 0.41	Control 6.99 ± 0.25	0.07/0.09
Cholecystocinin	3p22-p21.3	8,086,391	post-HSCT 5.61 ± 0.14	Control 5.89 ± 0.23	0.0014/0.003
GLP-1 receptor	6p21	8,119,338	post-HSCT 6.26 ± 0.08	Control 6.61 ± 0.27	0.0000/0.0001
FGF-21	19q13.1-qter	8,030,105	pre-HSCT 5.46 ± 0.15	Control 5.59 ± 0.16	0.0395/0.4325
			post-HSCT 5.36 ± 0.12	Control 5.59 ± 0.16	0.0009/0.0021

All the primary microarray data were submitted to GEO public repository and are accessible using GEO Series accession number GSE69421 (https://www.ncbi.nlm.nih.gov/geo/query/acc.cgi?acc=GSE69421). In our study a part of submitted microarray data was used.

### Statistical analysis

Continuous clinical and biochemical variables are presented as the mean values and standard error or as the median values and quartiles, as appropriate. Categorical variables are presented as frequencies and percentages. The Shapiro-Wilk test was used to assess the normal distribution of the continuous variables. To examine the differences between two or more independent groups, ANOVA/Student’s t-test (for variables with normal distribution) or Kruskal-Wallis/Mann-Whitney tests (for variables with non-normal distribution) were used. To assess the correlations between two continuous variables, Spearman rank correlation coefficient was calculated. The two-sided *p*-values < 0.05 were considered statistically significant. Gene expression data were RMA-normalized and presented as the mean and standard deviation. ANOVA was used to examine the differences in gene expression between two independent groups. The Benjamini-Hochberg (B-H)-corrected p-values < 0.05 were considered statistically significant. The statistical analyses were performed using the R 3.4.3 software.

### Ethical issues

The Permanent Ethical Committee for Clinical Studies of the Jagiellonian University Medical College approved the study protocol. All parents, adolescent patients, and adult patients signed a written informed consent before blood sample collection. Study was conducted in accordance with the Declaration of Helsinki.

## Results

When comparing the pre-HSCT and post-HSCT groups and the control group (Table 5), we noted a significantly lower BF mass and BF% measured using bioimpedance (6.46/12.0; 6.65/12.0, *p* < 0.05). The comparative analysis of the pre-HSCT group and the post-HSCT group showed no significant differences in anthropometric parameters.

**Table 5:** Values of adipose tissue parameters in studied groups and control

Parameter	pre-HSCT	post-HSCT	Control	***P*** value, pre-HSCT vs post-HSCT	P value, pre-HSCT vs control	***P*** value, Post-HSCT vs control
BMI^a^	18.9 (3.33)	18.3 (3.47)	19.1 (3.00)	0.173	0.794	0.405
BMIPerc^b^	70.4 [44.9;86.4]	51.0 [16.2;90.6]	77.7 [46.7;84.3]	0.170	0.967	0.486
BMISDS^a^	0.57 (1.21)	0.37 (1.26)	0.61 (0.87)	0.392	0.898	0.455
BF_kg^a^	6.46 (6.42)	6.65 (5.35)	12.0 (8.46)	0.854	0.031	0.029
BF_%^a^	14.5 (11.0)	15.8 (8.71)	21.1 (8.54)	0.616	0.042	0.062

^a^Mean values (standard deviations), paired Student Test for pre-HSCT vs. post-HSCT, and unpaired Student test for comparison with Control

^b^Medians [first and third quartile], Mann- Whitney test p-value

### Ghrelin

**Fig. 1:**
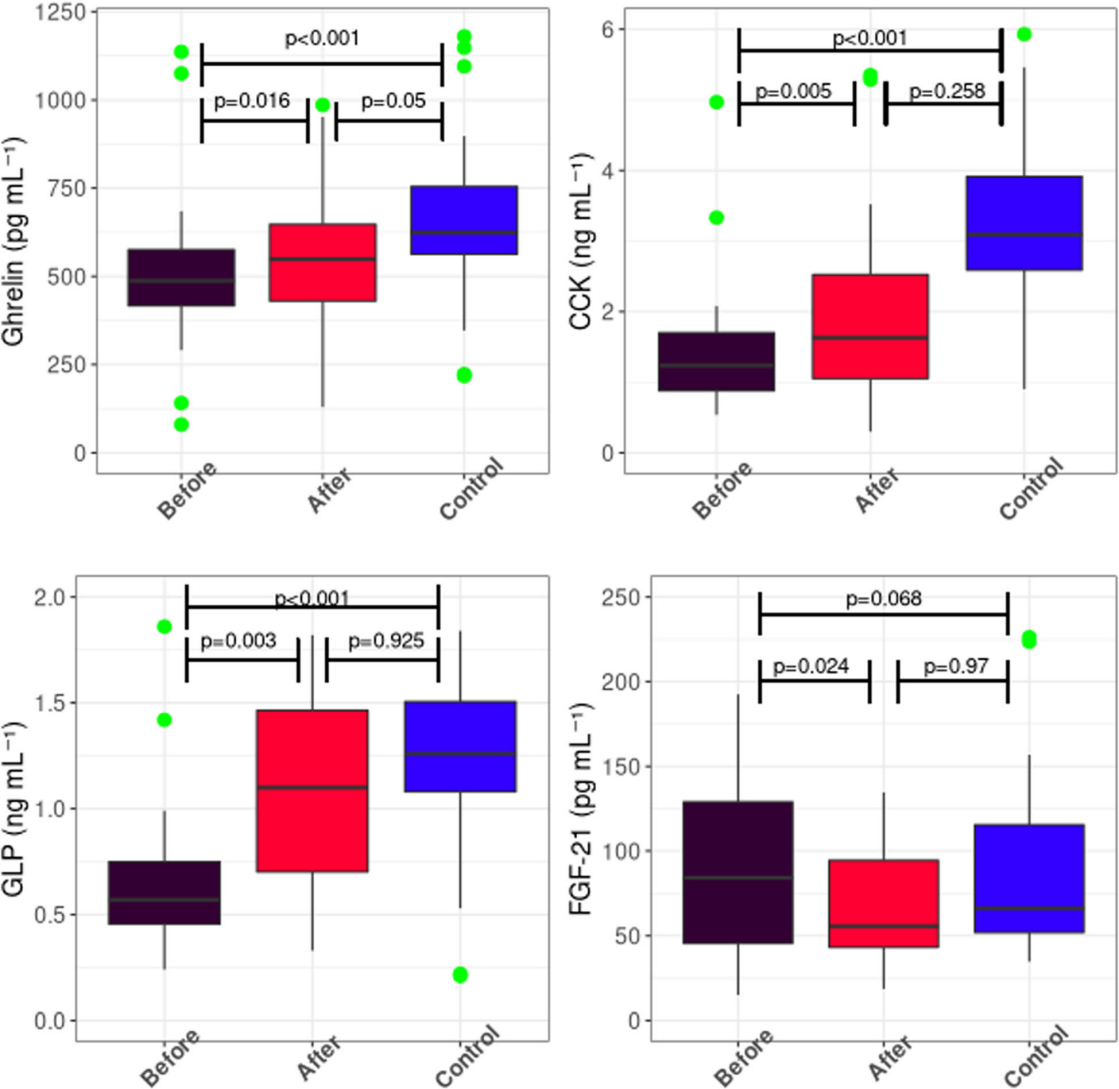
Boxplots of the distribution of the peptides. From left up: Ghrelin, Cholecystokinin (CCK), Glucagon like peptide-1 (GLP-1), Fibroblast growth factor-21 (FGF-21). *P*-values after Kruskall-Wallis or Mann-Whitney test are given above the corresponding boxes

The median ghrelin concentrations in the pre-HSCT group (median 501 pg/ml [first and third quartile 425;582]) and in post-HSCT group (558 pg/ml [445;701]) were significantly lower compared with the median concentration in the control group (711 pg/ml [596;898]) (*p* < 0.001 and *p* = 0.05, respectively). Differences in ghrelin concentrations between the pre-HSCT and post-HSCT groups were statistically significant (*p* = 0.016) (Fig. 1). Statistical analysis also revealed a considerable trend towards significance (*p* = 0.08) for the decreased ghrelin concentrations in patients with mucositis. Interestingly, ghrelin levels were increased in patients with liver aGvHD comparing with those with cutaneous and intestinal aGvHD (*p* = 0.02). Analysis of ghrelin gene expression revealed near-significantly (*p* = 0.07) lower (6.84+/− 0.41 vs. 6.99+/− 0.25) values in the post-HSCT group compared with the control group (Benjamini-Hochberg corrected *p*-value (B-H) = 0.09; Table 4).

### Cholecystokinin

Median CCK concentration in the pre-HSCT group (1.23 ng/ml; [first and third quartile 0.88;1.70]) was significantly lower than in the post-HSCT group (2.32 ng/ml [1.42;6.58]; *p* < 0.005) and in the control group (3.46 ng/ml [2.87;5.12]; *p* < 0.001). CCK concentrations in the post-HSCT group and control group showed no significantly differences (Fig. 1). The analysis of CCK gene expression revealed that mean CCK gene expression was significantly (*p* = 0.0014, B-H = 0.03) lower (5.61+/− 0.14 vs. 5.89+/− 0.23) in the post-HSCT group than in the control group (Table 4).

### Glucagon like peptide-1

The lowest median GLP-1 concentrations were seen in the pre-HSCT group (0.62 ng/ml [first and third quartile 0.47; 0.90]. The values observed in the post-HSCT group (1.31 ng/ml [0.83;1.82]) and in the control group were not significantly different (1.26 ng/ml [1.14;1.56]). The differences between the pre-HSCT group and the post-HSCT group, as well as between the pre-HSCT group and the control group, were significant (*p* < 0.003, p < 0.001 respectively; Fig. 1). Median concentration of GLP-1 was significantly decreased in patients with aGvHD symptoms (*p* = 0.008, Additional File 1). Moreover, GLP-1 levels negatively correlated with grade of aGvHD (r = − 0.58). Logistic regression model indicates that GLP-1 concentration may be a potential biomarker of aGvHD progression (*p* = 0.03).

GLP-1 receptor gene expression revealed a significantly lower mean expression (6.26+/− 0.08 vs. 6.61+/− 0.27) in the post-HSCT group compared with the control group (*p* = 0.000, B-H = 0.0001; Table 4).

### Fibroblast growth factor-21

Median FGF-21 concentrations seen in the pre-HSCT group (146 pg/ml; [first and third quartile 83.9; 303]) were higher than in the post-HSCT group (64.8 pg/ml [45.9;135]; *p* = 0.024) and in the control group (65.3 pg/ml [51.9;115]; *p* = 0.068). Analysis of FGF-21 gene expression revealed that its mean expression was significantly lower (5.36+/− 0.12 vs. 5.59+/− 0.16, *p* = 0.0009, B-H = 0.0021) in the post-HSCT group than in the control group (Table 4). No significant correlations between conditioning intensity or severity of mucositis grade and the studied peptide concentrations were found.

**Fig. 2:**
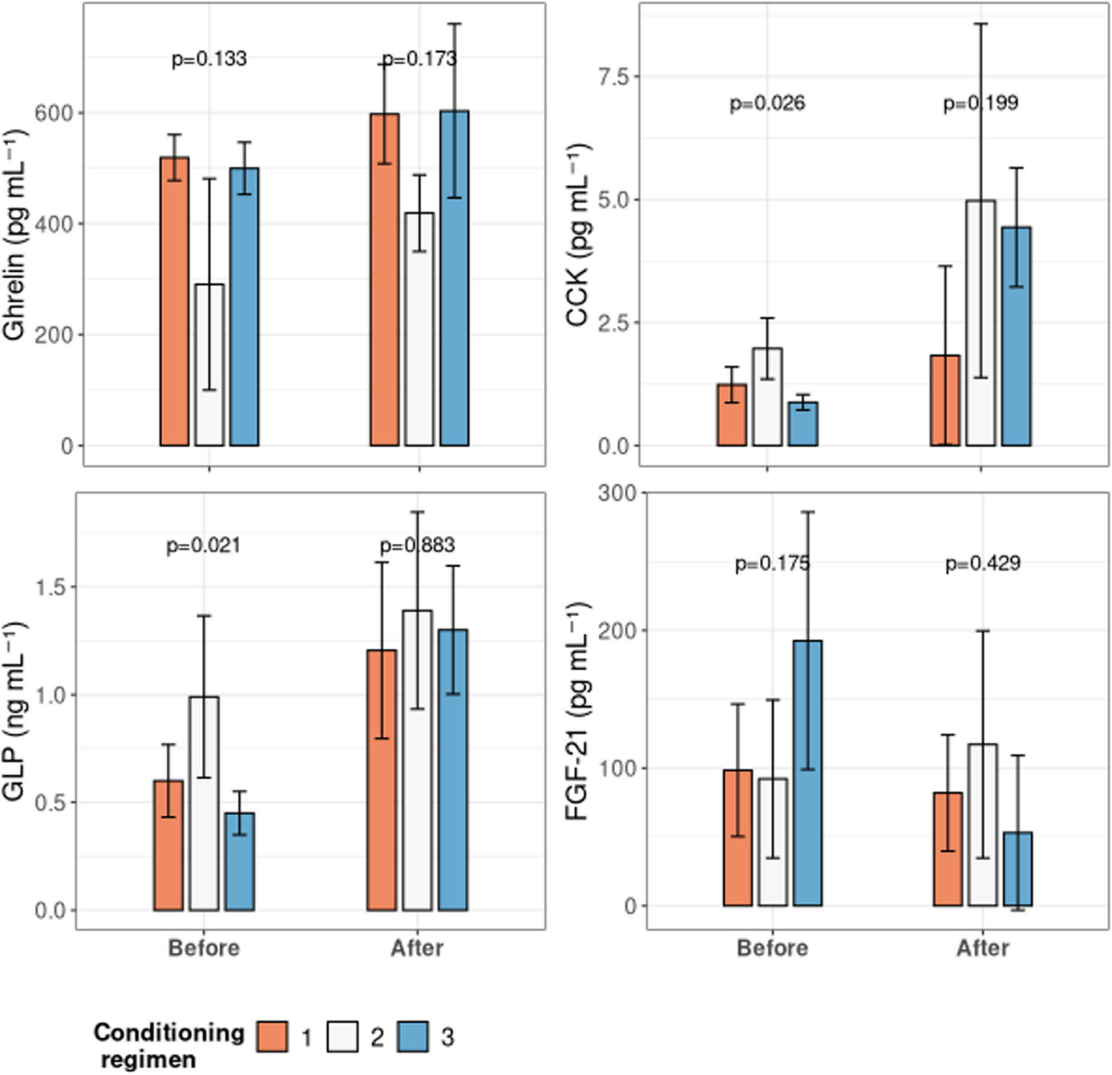
Median concentrations of peptides (bars) with standard error (lines) before and after HSCT depending on regime used for conditioning (BUS = Busulfan, CYC = Cyclofosphamide, TBI = total body irradiation). From left up: Ghrelin, Cholecystokinin (CCK), Glucagon like peptide-1 (GLP-1), Fibroblast growth factor-21 (FGF-21). P-values after Kruskal-Wallis test are given per group analyzed

No significant differences in the peptide levels were found between group with chemotherapy with Busulfan or Cyclophosphamide and TBI (Fig. 2).

## Discussion

Conditioning regimens are highly toxic to GI mucosal cells. The damage to the GI tract as well as other organs causes adverse effects, like nausea, vomiting, or diarrhea [21]. The effect of the treatment (chemotherapy and irradiation) of primary disease and effect of HSCT procedure cannot be easily distinguished. After the treatment of primary disease adverse effects are also observed. In our study 9 of 27 examined patients were without any previous treatment (Table 1). Before HSCT procedure significant difference was noted in CCK concentration in non- neoplastic disease group compare to neoplastic (median of 2.02 vs 1.07 ng/ml, *p* = 0.003). Same comparison 6 months after HSCT has shown significant difference in FGF-21 concentration in non-neoplastic disease group compare to neoplastic (median of 48.1 vs 114 pg/ml, *p* = 0.044, Additional File 2). Clinical symptoms of GI tract damage are well described, but there are no precise markers of advanced intestinal involvement and/or recovery. Endoscopic evaluation and intestinal biopsy are not recommended in patients with acute disease due the high risk of bleeding from seriously damaged mucosa and perforation. Therefore, there is a need to define blood biomarker that would correlate with location and severity of mucositis. Recently, serum citrulline (a non-essential amino acid) was proposed as a biomarker of small intestinal enterocyte mass and function [22, 23]. Citrulline indicates damage to the small intestine but is not specific to the intestinal enterocytes, because it is also produced in hepatocytes [24]. Therefore, we looked for other possible markers of GI mucositis dedicated to various levels of the gut. We studied concentrations of ghrelin produced in the stomach, CCK produced in the jejunum, GLP-1 produced in the ileum, and FGF-21 produced in the liver, pancreas, and white and brown adipose tissue [25, 26]. Cells of immune system are directly involved in acute graft versus host disease and they are infiltrating GI tract abundantly, therefore we hypothesized that this could be a cellular source, taking also into the account that gastrointestinal tract is one of the largest organs rich in lymphatic and vascular tissue itself. The expression of GI peptides has been investigated previously, not only in the gastrointestinal tract, but also in other tissue. For example ghrelin mRNA is naturally expressed in intestinal tissue but also in lymphocytes, neutrophils and lymphoid tissue [27]. Cholecytokinin was considered in some studies as a potential marker for Ewing Sarcoma in children [28]. GLPR-1 receptor is expressed on various immune cells and shows anti-inflammatory effects - decreasing proliferation of T- cells, increasing number of T regulatory cells [29]. To our knowledge only FGF-21 was underinvestigated in this matter. From the fact that expression of this peptides was significant in cells circulating in peripheral blood we can draw a conclusion that there is physiological relevance. There is little information in the literature on examined peptides in disease state, especially in metabolically unstable patients.

The amount of body fat did not influence peptide secretion, as the HSCT subgroups did not differ in terms of anthropometric parameters. Our study showed that 6 months after conditioning there was a significant increase in the secretion of ghrelin, CCK, and GLP-1. Plasma concentrations of these peptides were lower in the pre-HSCT group than the post-HSCT (convalescent) group and the control group. Kuruca et al. showed that irradiation during the treatment of intestinal cancers was associated with a decrease in ghrelin concentrations [30]. The low concentrations of ghrelin persisted 3 months after irradiation. Statistical analysis of our data revealed a considerable trend towards significance (*p* = 0.08) for the decreased ghrelin concentrations in patients with mucositis. Moreover, we found that 6 months after irradiation patients had higher levels of ghrelin compared to the values before conditioning. This suggests recovery of ghrelin secretion. This is favorable because ghrelin reduces intestinal injury and mortality after irradiation in animal models [31]. Interestingly, ghrelin levels were increased in patients with liver aGvHD compared with those with cutaneous or intestinal aGvHD (*p* = 0.02). This suggests dysregulation of gastric peptide secretion caused by liver damage.

CCK has anti-inflammatory properties and reduces cell apoptosis [32, 33]. We found higher concentrations of CCK after HSCT suggesting regeneration of the upper small intestine. The median concentration of GLP-1 was significantly decreased in patients with aGvHD symptoms. Moreover, GLP-1 levels negatively correlated with severity of aGvHD. In addition, GLP-1 concentrations returned to baseline (the values seen in healthy subjects) 6 months after conditioning. This suggests full recovery of the ileum. Logistic regression model indicates that GLP-1 concentration could be a potential biomarker for progression of aGvHD.

Increased concentrations of FGF-21 before conditioning suggest that hepatic injury may result from prolonged chemotherapy administered before HSCT. Animal models show that liver damage induces FGF-21 expression [34]. Conditioning adds to an additional liver injury. FGF-21is recognized as a stress response hepatokine that reduces hepatic damage through increased glucose uptake by adipose tissue. Normalization of FGF-21 concentrations 6 months after HSCT suggests complete recovery of hepatic function after transplantation. The FGF-21 gene expression data confirm the findings from biochemical analysis. Although we found statistically significant differences in peptide concentrations and gene expression model, the limitation of the current study is small sample size. On the other hand, examined group is unique. The presented results seem promising for establishing new diagnostic tools and provide the background for further investigation.

## Conclusions

Conditioning before HSCT and GvHD result in a widespread damage to the GI tract. Our data reveal that the stomach, jejunum, ileum, and liver are affected by chemo- and radiotherapy. Ghrelin may be a biomarker for liver aGvHD, and GLP-1 seems to be a potential biomarker for the progression of aGvHD. The increases in the concentrations of the regulatory peptides secreted in all parts of the GI tract suggest intensive regeneration of the mucosa. These alterations seem to be beneficial. The peptide measurements allow us to monitor intestinal damage and regeneration. Our study also showed that dysregulation of peptide secretion in some segments of the intestine are long-lasting, as 6 months after HSCT increased ghrelin secretion in the stomach, as well as CCK secretion in the jejunum, did not return to the values seen in the control group. The gene expression data are consistent with the biochemical data.

## Supplementary information

**Additional file 1: Supplementary Table 1.** Mean concentrations of peptides in post-HSCT group in aGvHD, mucositis and regarding localisation of aGvHD. Group *n* = 27. Freq = Frequency (%). *P*-values given after ANOVA test (*p* < 0.05).

**Additional file 2:Supplementary Table 2.** Median concentrations and quaritiles (in brackets) of peptides in treated group, patients with non- neoplastic and neoplastic disease before and after HSCT. P-values given after Kruskal-Wallis test.

## Data Availability

The datasets generated and/or analysed during the current study are available in the GEO public repository and are accessible using GEO Series accession number GSE69421 (https://www.ncbi.nlm.nih.gov/geo/query/acc.cgi?acc=GSE69421). All remaining datasets used and/or analysed during the current study are available from the corresponding author on reasonable request.

## Abbreviations

ALT: Alanine transaminase
aGvHD: Acute graft-versus- host disease
BF: Body fat
BMI: Body mass index
BMIPerc: BMI percentile
BMISDS: BMI standardised
BUS: Busulfan
CBC: Complete blood count
CYC: Cyclofosphamide
EIA: Enzyme immunoassay
FGF21: Fibroblast growth factor-21
CCK: Cholecystokinin
GI: Gastrointestional tract
GLP-1: Glucagone like peptide-1
GvHD: Graft-versus- host disease
HSCT: Hematopoietic stem cell transplantation
JMML: Juvenile myelomonocytic leukemia
TBI: Total body irradiation
