# Supplementary material for: Gastrointestinal peptides in children before and after hematopoietic stem cell transplantation
Source: BMC Cancer. 2020 Apr 15;20:306. doi: 10.1186/s12885-020-06790-9 (PMC7161205; doi:10.1186/s12885-020-06790-9)
Supplement: Supplementary file 1 — Additional file 1: Supplementary Table 1. Mean concentrations of peptides in post-HSCT group in aGvHD, mucositis and regarding localisation of aGvHD. Group n = 27. Freq = Frequency (%). P-values given after ANOVA test (p < 0.05). [file 12885_2020_6790_MOESM1_ESM.doc]

Additional File 1

Supplementary Table 1. Mean concentrations of peptides in post-HSCT group in aGvHD, mucositis and regarding localisation of aGvHD. Group n=27. Freq= Frequency (%). P-values given after ANOVA test (p<0.05).
