# Supplementary material for: Gastrointestinal peptides in children before and after hematopoietic stem cell transplantation
Source: BMC Cancer. 2020 Apr 15;20:306. doi: 10.1186/s12885-020-06790-9 (PMC7161205; doi:10.1186/s12885-020-06790-9)
Supplement: Supplementary file 2 — Additional file 2:Supplementary Table 2. Median concentrations and quaritiles (in brackets) of peptides in treated group, patients with non- neoplastic and neoplastic disease before and after HSCT. P-values given after Kruskal-Wallis test. [file 12885_2020_6790_MOESM2_ESM.doc]

Additional File 2

Supplementary Table 2. Median concentrations and quaritiles (in brackets) of peptides in treated group, patients with non- neoplastic and neoplastic disease before and after HSCT. P-values given after Kruskal-Wallis test.
